# Awareness of Biomedical Waste Management in Dental Students in Different Dental Colleges in Nepal

**DOI:** 10.1155/2018/1742326

**Published:** 2018-12-09

**Authors:** Tanuja Singh, Tika R. Ghimire, Santosh K. Agrawal

**Affiliations:** ^1^Department of Dentistry, Devdaha Medical College and Research Institute, Rupandehi, Nepal; ^2^Department of Community/Public Health Dentistry, CODS, BPKIHS, Dharan, Nepal

## Abstract

**Aim:**

The aim of this study is to assess the awareness of biomedical waste management in dental students of various dental colleges of Nepal.

**Methodology:**

A structured pretested questionnaire was used among 434 (323 males and 111 females) undergraduate dental students of five different dental colleges of Nepal. First part of the questionnaire was used to describe demographic profile of the participants. Second part of the questionnaire assessed the knowledge, attitude, and practice regarding biomedical waste management. Chi-square test was applied to find out the association between different responses obtained from different colleges.

**Result:**

Majority (91.82%) of participants had a positive attitude towards safe management of biomedical waste. Regarding the knowledge of BMW management policies, majority of the students (83.1% to 98.9%) had positive attitude towards the safe management of biomedical waste, whereas more than 50% of the students were unaware of the guidelines laid down by Government of Nepal. Regarding biomedical waste disposal technique in the hospital, only 29.9% to 79.8% are aware; this shows that there is lack of strict protocol in the BMW management. Association between different responses and colleges for “improper waste management causes various health hazards” ranged from 93.3% to 98.9%.

**Conclusion:**

There exists a lacuna in the knowledge and practice of biomedical waste management among the undergraduate dental students in Nepal. Since the students had positive attitude towards addressing this concern, workshops and trainings related to proper biomedical waste management would be a step forward towards attaining a healthy environment for the future.

## 1. Introduction

With the civilization and advancement in the medical technology, a greater population is having access to health services than before [1]. The increased accessibility of healthcare facilities has not only significantly improved quality of life of population but also threatens the community health due to production of tremendous amount of biomedical waste. Biomedical waste is defined as “any solid, fluid, or liquid waste, including its container and any intermediate product, which is generated during diagnosis, treatment, or immunization of human beings or animals, in research pertaining thereto or in the production or testing of biological and animal waste from slaughter houses or any other like establishments” [2]. Dental waste is a subset of hazardous biomedical waste (BMW). It includes various materials like soaked cotton, sharp needles, extracted teeth, human tissue parts, and so forth, which are usually contaminated with body fluids like blood and saliva [3]. Dental practices also produce a few other types of waste, such as mercury, silver amalgam, and various chemical solvents [4]. If the manipulation of amalgam and its waste products are not strictly regulated, it could be responsible for environmental pollution as well as occupational exposure [5]. Dental waste can also have two types of effects, that is, on the environment and on the health of the person handling the waste [6].

With rapid increase in the number of healthcare institutions in Nepal, the burden of biomedical waste generated is also increasing [7]. Environment and Public Health Organization, Nepal, has reported an average healthcare waste generation of 1.7 kg/person/day and 0.48 kg/person/day of Healthcare Risk Waste (HCRW) at an average bed occupancy rate of around 65% [8]. Lack of proper knowledge and appropriate technique of handling of biomedical waste can lead to serious consequences on health and environment.

Every institution has guidelines and protocol for management of biomedical waste. These guidelines and protocols should strictly be followed at every level of generation, collection, transportation, storage, treatment, and disposal. At the level of generation itself, biomedical waste should be segregated into color-coded bags or containers. A proper mechanism should be developed to collect, transport, store, or dispose such hazardous waste to avoid serious public health consequences. All those involved in different levels from generation to disposal are potentially at risk of serious health consequences. The risk group includes doctors, nurses, auxiliaries, hospital staffs, and workers handling and disposing such waste [9]. Healthcare facilities should implement strict rules and regulations with proper training to staffs [10]. Due to laxity in implementation of the rules and inadequate training to healthcare personnel, there is an indiscriminate disposal of biomedical waste [11].

In dental school setup, students are involved in clinical activities that include generation of biomedical waste. It is essential that they have sound knowledge about guidelines and practice of biomedical waste management protocol. In context of Nepal, very few studies have been done on BMW management. Although many studies have been carried out among medical professionals [12, 13], no study has been carried out to assess knowledge, attitude, and practices of biomedical waste management involving students studying dentistry in Nepal [14]. Thus the purpose of the study was to assess the knowledge and practice of biomedical waste management among students of various dental colleges.

## 2. Materials and Methods

A descriptive, cross-sectional study was carried out among undergraduate dental students pursuing dentistry in their third, fourth, and final years as well as interns of five different dental colleges in Nepal. All the students in their clinical years who consented for the study were included. Total duration of the study was six months, from July 2017 to December 2017. The study was carried out after obtaining required clearance from the Institutional Review Committee of the Kathmandu University (IRC-KUSMS, protocol approval no. 91/17). For each institute, different code (A, B, C, D, and E) was given to maintain the confidentiality. Sampling was done based on convenience method to include maximum number of students. A total of 500 questionnaires were distributed, where only 434 students returned them with complete answers, including 111 males and 323 females.

Data collection was done with the help of a structured close-ended questionnaire (*see questionnaire *available here) that consisted of two parts. The first part of the questionnaire consisted of questions for demographic profile of the participants, while the second part assessed the awareness and practice on biomedical waste management with seventeen questions. Of the seventeen questions, the first three questions assessed students' knowledge and attitude regarding BMW management policies. The next nine questions assessed the knowledge on BMW management practices and the last five questions evaluated the participants' awareness and education regarding BMW management. The questionnaire was administered to the participants by the author with proper instructions.

Master chart and coding list was prepared before entering the data and then the collected data was entered into the computer through Microsoft Excel Sheet. Data was transferred to SPSS for statistical analysis. For statistical analysis, Chi-square test was applied to find out the association between different responses obtained from different colleges. P value ≤ 0.01 was considered statistically significant.

## 3. Results

**Figure 1:**
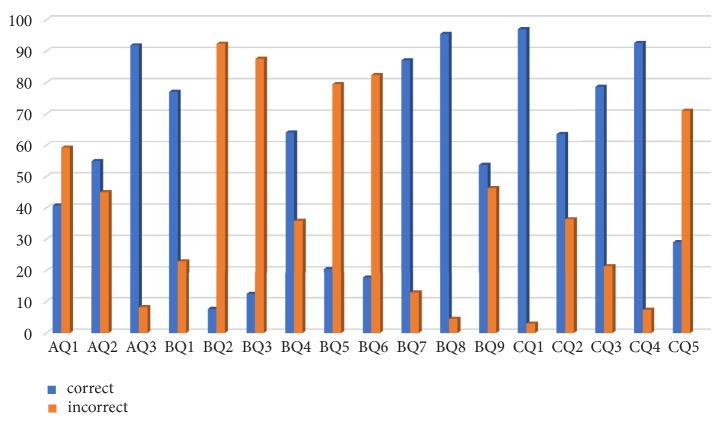
Bar diagram representation of correct and incorrect answer for the questionnaire.

A total of 434 completely filled questionnaires were obtained (111 male and 323 female participants) from five different dental colleges of Nepal. Majority (91.82%) of participants had a positive attitude towards safe management of biomedical waste. The correct responses ranged from 83.1% to 98.9% (Figure 1). Statistically significant difference was found in BMW management policy and safe disposal of waste among different colleges students (p<0.001). Meanwhile, no difference was found in knowledge regarding government guidelines on waste management (p=0.217) (Table 1).

Most (92.3%) of the participants lacked knowledge regarding disposal of used plastic items (e.g., suction tubes) in different colored bags and difference was statistically significant (p≤0.001). Majority (95.5%) of them agreed to wearing gloves and mask while handling BMW. In one of the institutions, all the students were adopting the use of protective barriers and difference was statistically significant (p≤0.001). Correct response rate for disposal of excess mercury in air tight container ranged from 80% to 89.7%. However, the difference was not significant among different colleges (p=0.408).

Correct response rate for the “different color bag to dispose different types of waste” was highest in institution C, whereas incorrect rate was highest in college D, 82% and 27.1%, respectively. The question about “used sharps and needles are disposed in” was correctly answered by different institute's students ranging from 48.3% to 84.3% with significant difference among them (p value<0.001). Practice of “treating infectious waste before disposing” ranges from 39.3% to 65.9%, which was statistically significant (p≤0.001) (Table 2).

In the education and awareness of biomedical waste management part, almost all students concurred that biomedical waste causes health hazards. The correct response ranged between 93.3% and 98.9% and no statistical difference was found (p=0.268). Likewise, no statistical difference was found in knowledge assessment question “Does your hospital/clinic generate biomedical waste?” among different colleges (p=0.905). On average, 92.6% (range: 79.8% to 97.9%) of students agreed that there should be regular educational programmes on biomedical waste management. However, only 29% (range: 11.4% to 46.1%) of students accepted to receive training in any form (e.g., lecture and workshop) on BMW, which was statistically significant with p value < 0.001 (Table 3).

## 4. Discussion

Proper management of biomedical wastes involves active involvement and synchronization between governmental and nongovernmental bodies, the medical institutions, and the healthcare personnel. In developing nations like Nepal, strong rules and regulation for the segregation and appropriate management of BMW may be lacking [15]. Many studies regarding BMW management from developing countries like India [16, 17], Brazil [18], Bangladesh [19], and Turkey [20] documented in the literature showed inadequate knowledge and indifferent attitude among healthcare workers. Dental waste is also a component of biomedical waste but, due to its catastrophic, chemical, and contagious contents, its safe management is very complex. As dental students are future dentists of the country, assessing their knowledge and awareness regarding BMW helps us to locate where the necessary changes can be done for the proper implementation of the policies regarding BMW management.

The cross-sectional study was conducted on predesigned and pretested questionnaire with the objective which analyzes the knowledge and attitude regarding BMW management policies, practices, and awareness among the dental students. Since we have not found any study in Nepal addressing the same objectives, key strength of this study was that this assessment of awareness of BMW management in dental students in different dental colleges in Nepal will provide us a unique opportunity to provide information about a topic that is lacking in our country and also help to include this topic in their curriculum.

In BMW policies, majority of the students, 83.1% to 98.9%, have positive attitude towards safe management of biomedical waste, whereas less than 50% of students are aware of the guidelines laid down by Government of Nepal for BMW management. Regarding biomedical waste disposal technique in the hospital, only 29.9% to 79.8% are aware; this shows that there is lack of strict protocol in the BMW management. A functional guideline has been proposed under the National Healthcare Technology strategy on second health plan (1997-2017) to manage all the medical waste including waste generated from private sector, for which strict implementation is required for this initiative, which should be taken from the student level itself [7].

Regarding BMW practices among students of various dental institutions, maximum awareness was found regarding disposal of mercury (79.8%-97.9%), which is considered to be a major environmental hazard [20]. This can be attributed to the detailed description of dental amalgam in the subject of dental materials, which is taught during the preclinical years of a dental curriculum. The American Dental Association approved that excess amalgam in a small amount should be stored in the “photographic fixer” in a closed container to minimize its hazard and then can be sent for recycling [21]. The students were aware of using protective barrier while handling BMW (75.7%-82.0%) followed by use of different color-coded bags for different types of waste and disposal of sharps and needles. This shows that students were aware of BMW management generated in day-to-day dental practices which require special attention, as they are health hazard items. But only 37.1%-65.2% of students knew about treating BMW before disposing them. Also, the knowledge regarding segregation of different wastes like plastic items, soiled dressing, impression material, extracted teeth, human tissue, and plaster of Paris in color-coded bags was inadequate. The information regarding effective disposal of other dental materials is lacking in the curriculum.

Our study highlights the fact that students of various dental institutions of Nepal have general awareness for the need to properly dispose biomedical waste but they are not exactly aware as how to do it effectively. Improper segregation and disposal of BMW and mixing it with municipal waste can result in possible exposure of the healthcare workers, waste handlers, waste pickers, and the general public to the microorganisms [9] and harmful chemicals which are responsible for highly infectious and fatal diseases. Infectious diseases contribute a major share to the burden of diseases resulting in high morbidity and mortality rates in developing countries [22]. A major share of these infections can be attributed to improper handling of biomedical wastes. Infections like HIV, Hepatitis, Tuberculosis, and others have a potential to spread through biomedical wastes [23, 24]. More than 50% of government institutions in Nepal do not practice waste segregation, is are relatively efficient in private institutions [25].

Lastly, on the topic of the knowledge and attitude regarding BMW awareness among students of various dental institutions, many are aware about improper waste management causing various health hazards (93.3%-98.9%), importance of regular educational programs on biomedical waste management (79.8-97.9%), hospital/clinic generate biomedical waste (75.7%-82.0%), and maintaining BMW records mandatory in the hospital/clinic (52.8%-87.6%). But only a few have received training in any form (e.g., lecture and workshop) on BMW management (11.4%-46.1%). Nepal Health Research Council (NHRC) has published two booklets, namely, National Healthcare Waste Management Guidelines and Healthcare Waste Management-Training Manual, for medical professionals to implement BMW management guidelines in their healthcare establishment, which is a good initiative [26].

The results of this study showed that the dental students were well aware that the dental hospitals/clinics generate BMW and improper waste management can cause various health hazards. Although their answers indicated that different types of biomedical waste should be disposed in different colored bags, very few are aware of which colored bags were for which forms of BMW. The findings of the present study suggest that proper waste management educational program should be included in the curriculum for dental education so as to give due importance to this vital issue. BMW management cannot be achieved successfully without the effective awareness, knowledge, motivation, education, and cooperation from all the healthcare employees.

## 5. Conclusion

The results of this study show that the dental students of various dental colleges in Nepal are well aware that hospitals and clinics generate hazardous waste and it should be disposed properly. But, on practice, participants lacked proper knowledge on guidelines for disposing specific type of waste. It is the need of the hour that the various universities providing dental education take this matter seriously and incorporate sufficient education materials about biomedical waste management in the curriculum and provide necessary trainings to reduce the overall burden of infectious diseases in a developing country like Nepal.

## Figures and Tables

**Table 1:** Quantitative analysis of the knowledge and attitude regarding BMW management policies among students of various dental institutions.

Question		Inst. A	Inst. B	Inst. C	Inst. D	Inst. E	Total %	X^2^	p
AQ1	Correct	42	41	38	29	27	40.78%	5.770	0.217
		(47.2%)	(42.3%)	(42.7%)	(41.4%)	(30.3%)			
	Incorrect	47	56	51	41	62	59.22%		
		(52.8%)	(57.7%)	(57.3%)	(58.6%)	(69.7%)			

AQ2	Correct	53	29	71	33	50	54.96%	49.127	<0.001
		(61.8%)	(29.9%)	(79.8%)	(47.1%)	(56.2%)			
	Incorrect	36	68	18	37	39	45.04%		
		(38.2%)	(70.1%)	(20.2%)	(52.9%)	(43.8%)			

AQ3	Correct	87	88	88	62	74	91.82%	20.380	<0.001
		(97.8%)	(90.7%)	(98.9%)	(88.6%)	(83.1%)			
	Incorrect	2	9	1	8	15	8.18%		
		(2.2%)	(9.3%)	(1.1)	(11.4%)	(16.9%)			

**Table 2:** The knowledge and attitude on BMW practices among students of various dental institutions.

Question		Inst. A	Inst. B	Inst. C	Inst. D	Inst. E	Total %	X^2^	p
BQ1	Correct	69	72	73	51	70	77.06%	2.524	0.640
		(77.5%)	(74.2%)	(82%)	(72.9%)	(78.7%)			
	Incorrect	20	25	16	19	19	22.94%		
		(22.5%)	(25.8%)	(18%)	(27.1%)	(21.3%)			

BQ2	Correct	6	3	9	1	15	7.64%	17.813	0.001
		(6.7%)	(3.1%)	(10.1%)	(1.4%)	(16.9%)			
	Incorrect	83	94	80	69	74	92.36%		
		(93.3%)	(96.9%)	(89.9%)	(98.6)	(83.1%)			

BQ3	Correct	11	6	14	12	11	12.4%	5.790	0.215
		(12.4)	(6.2%)	(15.7%)	(17.1%)	(12.4%)			
	Incorrect	78	91	75	58	78	87.6%		
		(87.6)	(93.8%)	(84.3%)	(82.9%)	(87.6)			

BQ4	Correct	51	58	75	51	43	64.1%	30.255	<0.001
		(57.3%)	(59.8%)	(84.3%)	(72.9%)	(48.3%)			
	Incorrect	38	39	14	19	46	35.9%		
		(42.7%)	(40.2%)	(15.7%)	(27.1%)	(51.7%)			

BQ5	Correct	22	16	22	19	10	20.5%	9.479	0.050
		(24.7%)	(16.5%)	(24.7%)	(27.1%)	(11.2%)			
	Incorrect	67	81	67	51	79	79.5%		
		(75.3%)	(83.5%)	(75.3%)	(72.9%)	(88.8%)			

BQ6	Correct	15	5	20	15	21	17.6%	15.410	0.005
		(16.9%)	(5.2%)	(22.7%)	(21.4%)	(23.6%)			
	Incorrect	74	92	68	55	68	82.4%		
		(83.1%)	(94.8%)	(77.3%)	(78.6%)	(76.4%)			

BQ7	Correct	79	87	78	56	78	87.1%	3.985	0.408
		(88.8%)	(89.7%)	(87.6%)	(80.0%)	(87.6%)			
	Incorrect	10	10	11	14	11	12.9%		
		(11.2%)	(10.3%)	(12.4%)	(20.0%)	(12.4%)			

BQ8	Correct	89	94	84	65	83	95.5%	7.251	0.046
		(100%)	(96.9%)	(94.4%)	(92.9%)	(93.3%)			
	Incorrect	0	3	5	5	6	4.5%		
		(0%)	(3.1%)	(5.6%)	(7.1%)	(6.7%)			

BQ9	Correct	54	36	58	36	49	53.7%	17.393	0.002
		(60.7%)	(37.1%)	(65.2%)	(51.4%)	(55.1%)			
	Incorrect	35	61	31	34	40	46.3%		
		(39.3%)	(62.9%)	(34.8%)	(48.6%)	(44.9%)			

**Table 3:** Quantitative analysis of the knowledge and attitude on BMW awareness among students of various dental institutions.

Question		Inst. A	Inst. B	Inst. C	Inst. D	Inst. E	Total %	X^2^	p
CQ1	Correct	88	95	86	69	83	97.0%	6.297	0.268
		(98.9%)	(97.9%)	(96.6%)	(98.6%)	(93.3%)			
	Incorrect	1	2	3	1	6	3.0%		
		(1.1%)	(2.1%)	(3.4%)	(1.4%)	(6.7%)			

CQ2	Correct	54	60	78	37	47	63.6%	30.640	<0.001
		(60.7%)	(61.9%)	(87.6%)	(52.9%)	(52.8%)			
	Incorrect	35	37	11	33	42	36.4%		
		(39.3%)	(38.1%)	(12.4%)	(47.1%)	(47.2%)			

CQ3	Correct	73	76	69	53	70	78.6%	1.030	0.905
		(82.0%)	(78.4%)	(77.5%)	(75.7%)	(78.7%)			
	Incorrect	16	21	20	17	19	21.4%		
		(18.0%)	(12.6%)	(22.5%)	(24.3%)	(21.3%)			

CQ4	Correct	86	95	87	63	71	92.6%	31.749	<0.001
		(96.6%)	(97.9%)	(97.8%)	(90.0%)	(79.8%)			
	Incorrect	3	2	2	7	18	7.4%		
		(3.4%)	(2.1%)	(2.2%)	(10.0%)	(20.2%)			

CQ5	Correct	34	18	41	8	25	29.0%	31.901	<0.001
		(38.2%)	(18.6%)	(46.1%)	(11.4%)	(28.1%)			
	Incorrect	55	79	48	62	64	71.0%		
		(61.8%)	(81.4%)	(53.9%)	(88.6%)	(71.9%)			

## Data Availability

The data used to support the findings of this study are available through the following URL: https://figshare.com/s/5422fa2d3ba5f8854ecd.

## Supplementary Materials

Supplementary Materials The questionnaire section has been removed from the main manuscript section and has been submitted as a supplementary file.
