# Supplementary material for: Awareness of Biomedical Waste Management in Dental Students in Different Dental Colleges in Nepal
Source: Biomed Res Int. 2018 Dec 9;2018:1742326. doi: 10.1155/2018/1742326 (PMC6304656; doi:10.1155/2018/1742326)
Supplement: Supplementary Materials — The questionnaire section has been removed from the main manuscript section and has been submitted as a supplementary file. [file 1742326.f1.pdf]

SUPPLEMENTARY FILE

QUESTIONNAIRE

Awareness of Biomedical Waste Management of In Dental Students In Different Dental Colleges In Nepal

Approximated time: 10 minutes

Socio- demographic profile of the respondent:

1. Age : ……………years   Date: /   /
2. Gender: a) Male b) Female
3. Level of education/Year of study ……………..

Note: Please read the following questions thoroughly and put a tick mark in the option corresponding to your choice.

A. BMW management policies

1. Are there any guidelines laid down by Government of Nepal for BMW management?
   a) Yes b) No c) Don't Know
2. Is there any biomedical waste disposal policy in your hospital/clinic?
   a) Yes b) No c) Don't Know
3. Safe management of biomedical waste is the:
   a) Responsibility of only government
   b) Team work of dental surgeons & auxiliaries
   c) Don't know

B. BMW management practices

1. Are different colored bags used to dispose different types of waste?
   a) Yes b) No c) Don't Know
2. Used disposable plastic items (e.g., Suction tubes) are disposed of in?
   a) Yellow bags b) Red bags c) Black bags d) Don't know

3. Soiled dressings and used impression materials are disposed of in:
   a) Blue/white bags b) Red bags c) Black bags d) Don't know
4. Used sharps and needles are disposed of in:
   a) Yellow bags b) Rigid/puncture-proof container
   c) Red bags d) Don't know
5. Extracted teeth and human tissue are disposed of in:
   a) Yellow bags b) Red bags c) Black bags d) Don't know
6. Plaster of Paris is disposed of in:
   a) Yellow bags b) Red bags c) Black bags d) Don't know
7. Excess mercury are disposed of in:
   a) Sink b) Air tight Containers c) Left on the tray d) Don't know
8. Do you use protective barriers (e.g. gloves, masks) during handling of BMW?
   a) Yes b) No
9. Do you treat infectious waste before disposing them?
   a) Yes b) No

C. Participants education/awareness

1. Can improper waste management cause various health hazards?
   a) Yes b) No c) Don't know
2. Is maintaining BMW records mandatory in your hospital/clinic?
   a) Yes b) No c) Don't know
3. Does your hospital/clinic generate biomedical waste?
   a) Yes b) No c) Don't know
4. Should there be regular educational programs on biomedical waste management?
   a) Yes b) No c) Don't know
5. Have you ever received training in any form (e.g. lecture, workshop) on BMW management?
   a) Yes b) No
